# Silent Jaw Expansion: A Rare Case of Posterior Maxillary Ameloblastoma

**DOI:** 10.7759/cureus.69165

**Published:** 2024-09-11

**Authors:** Nur Nadia Abd Rahim, Shamsul Anuar Ahmad, Herni Talib

**Affiliations:** 1 Otorhinolaryngology - Head and Neck Surgery, KPJ Healthcare University, Nilai, MYS; 2 Oral and Maxillofacial Surgery, KPJ Tawakkal Health Centre, Kuala Lumpur, MYS; 3 Histopathology, Lablink Medical Laboratory, Kuala Lumpur, MYS

**Keywords:** ameloblastoma, asian, maxillary ameloblastoma, maxillectomy, maxillofacial, odontogenic, posterior maxilla, surgery, surveillance, symptoms

## Abstract

Ameloblastoma is a rare odontogenic tumour that develops from the epithelial remains of the dental lamina. It is a benign but locally aggressive neoplasm that typically manifests as slow-growing tumours in the jaw, with posterior maxilla ameloblastoma being the rarer occurrence compared to mandibular ameloblastoma. This case report is about a 46-year-old Malay man who presented with a two-month history of left palatal and cheek swelling, along with symptoms such as left eye blurring, nasal block, and dysphagia. Imaging revealed a massive cystic lesion in the left maxillary sinus, which was diagnosed as a multicystic ameloblastoma after biopsy. The patient underwent surgical resection with a partial maxillectomy and was treated postoperatively with antibiotics, steroids, and regular follow-up appointments. The histopathological examination confirmed the diagnosis, and the patient was given a dental obturator to cover the defect intraorally. He is currently under annual surveillance with no signs or symptoms of recurrence.

## Introduction

Rarely are we able to find evidence of diseases dating back to the Cretaceous period, between 67 and 69 million years ago. However, a dinosaur fossil of a *Telmatosaurus *found in Romania revealed an expansile jaw tumour that had the characteristic of multilobulated cysts or mixed cystic and solid areas with cortical thinning, suggesting a diagnosis of ameloblastoma [1]. Fast forward to 1885, when Malassez first defined ameloblastoma and proposed that it arises from epithelial remnants of the developing root sheath [2, 3]. It wasn’t until 45 years later that Ivey and Churchill coined the term ameloblastoma, which came from the words ‘amel’ in English, which means enamel, and ‘blastos’ in Greek, which means the germ [4].

Ameloblastoma belongs to the odontogenic tumour group, which WHO classified into five main types in 2017 based on genetic studies [5]. The most updated classification divides ameloblastoma into i) ameloblastoma (including conventional, solid/multicystic, and intraosseous ameloblastoma); ii) ameloblastoma-unicystic type; iii) ameloblastoma-extraosseous/peripheral type (including soft tissue, mucosal, or gingiva origin); and iv) metastasising ameloblastoma. It is a benign but aggressive odontogenic neoplasm originating from the epithelial remnants of the dental lamina. The initial mutation that initiated the events leading to malignant transformation is the current accepted etiopathogenesis of malignant ameloblastoma. Further research is needed to identify the exact sequence of events and their pathogenesis. It has been found that genetic mutations that affect the mitogen-activated protein kinase (MAP-K) pathway mostly involve the BRAF gene, among others. Other known genes affecting the MAP-K pathway are KRAS, NRAS, HRAS, and FGFR [6]. Addressing this gap in knowledge will enable more targeted treatment for patients, especially those with unresectable tumours. 

## Case presentation

**Figure 1:**
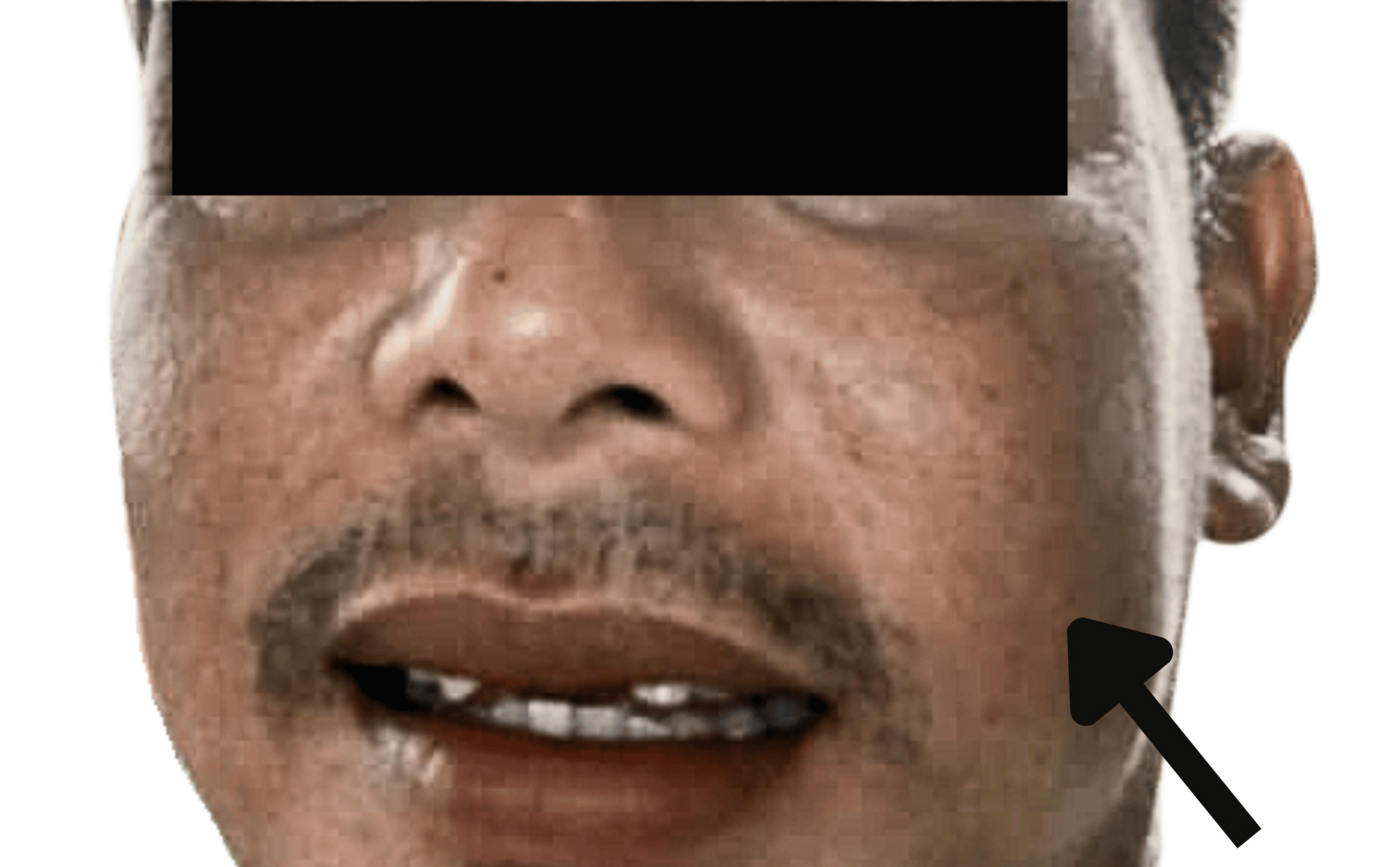
Diffuse swelling of the left side of the face (black arrow)

**Figure 2:**
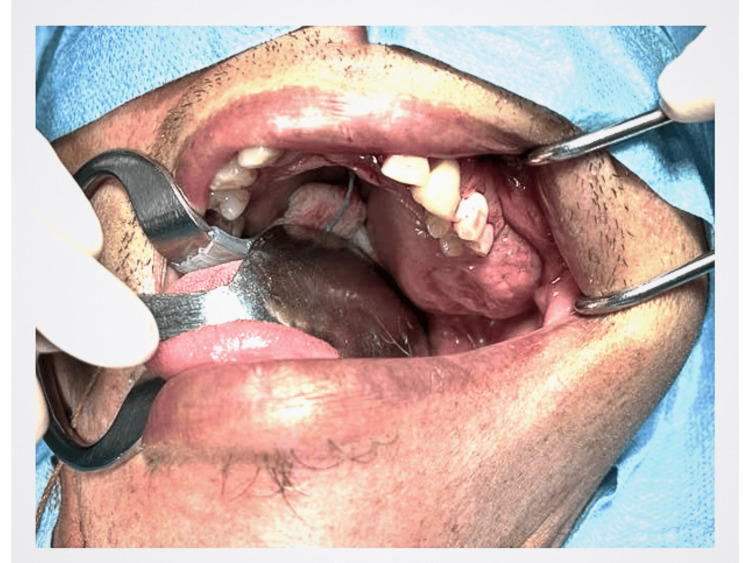
Left cystic palatal swelling is noted.

A 46-year-old gentleman of Malay origin presented with a history of worsening left palatal swelling and left cheek swelling for two months. It was associated with a blurring vision of the left eye, left nasal block, loosening left upper molars, left upper gum swelling, difficulty chewing, altered sensation over the left palatal mass, and itchiness of the skin overlying the cheek. There was no medical or surgical history noted. The patient worked as a labourer and had a smoking history of 30 pack-years. There was no family history of head and neck malignancy. Physical examination revealed a diffuse swelling over the left cheek without any skin changes, as shown in Figure 1. There was no left eye proptosis or any impaired sensation over the left cheek. A left cystic palatal swelling measuring 3 x 3 cm with no mucosal ulceration was noted in the intraoral examination with no breach of the retromolar trigone (Figure 2). Differential diagnoses included benign maxillary tumours or cervicofacial vascular malformation [7].

**Figure 3:**
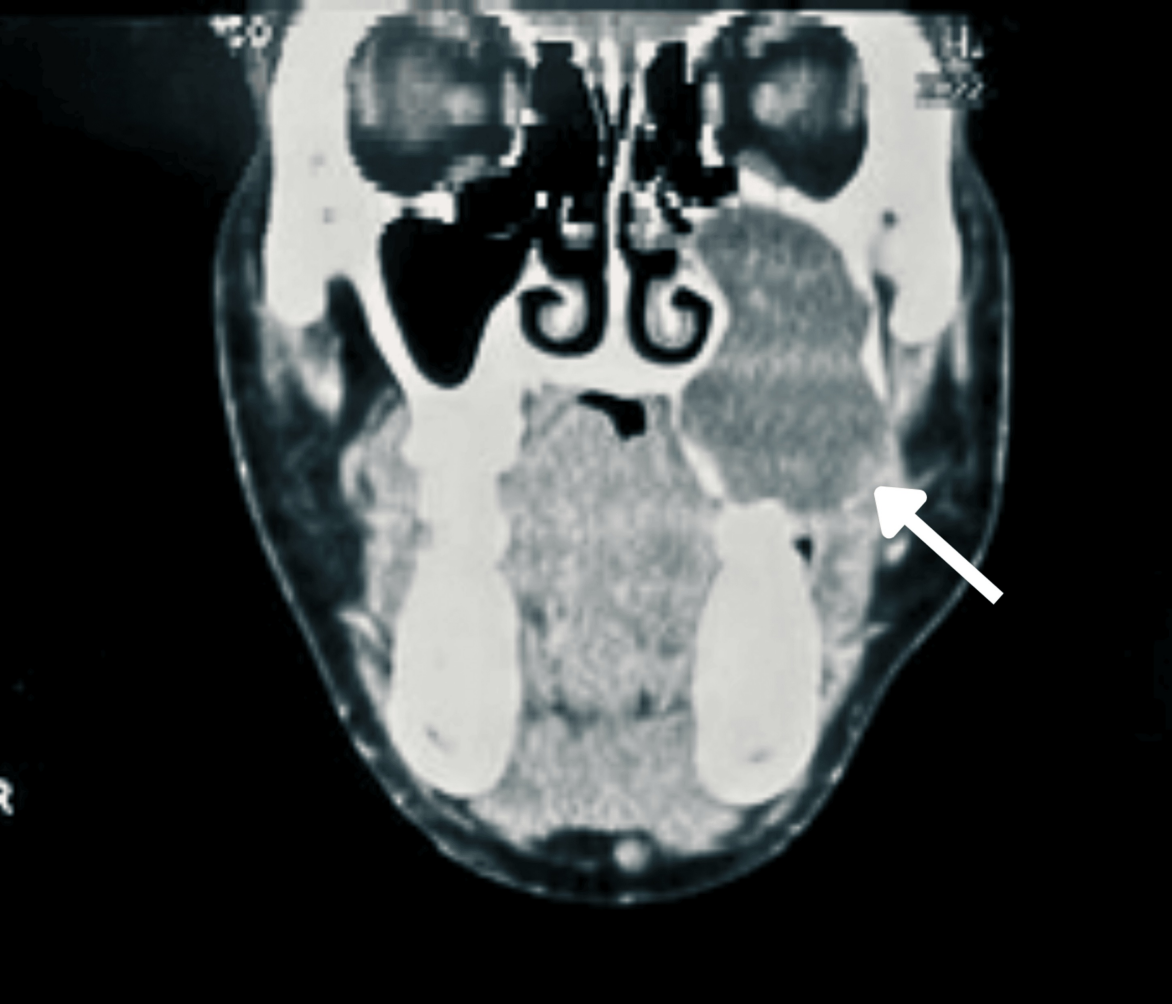
The CT scan (coronal view) shows a lesion occupying the left maxillary sinus and cortical defects over the inferior aspect of the left maxillary sinus (white arrow).

**Figure 4:**
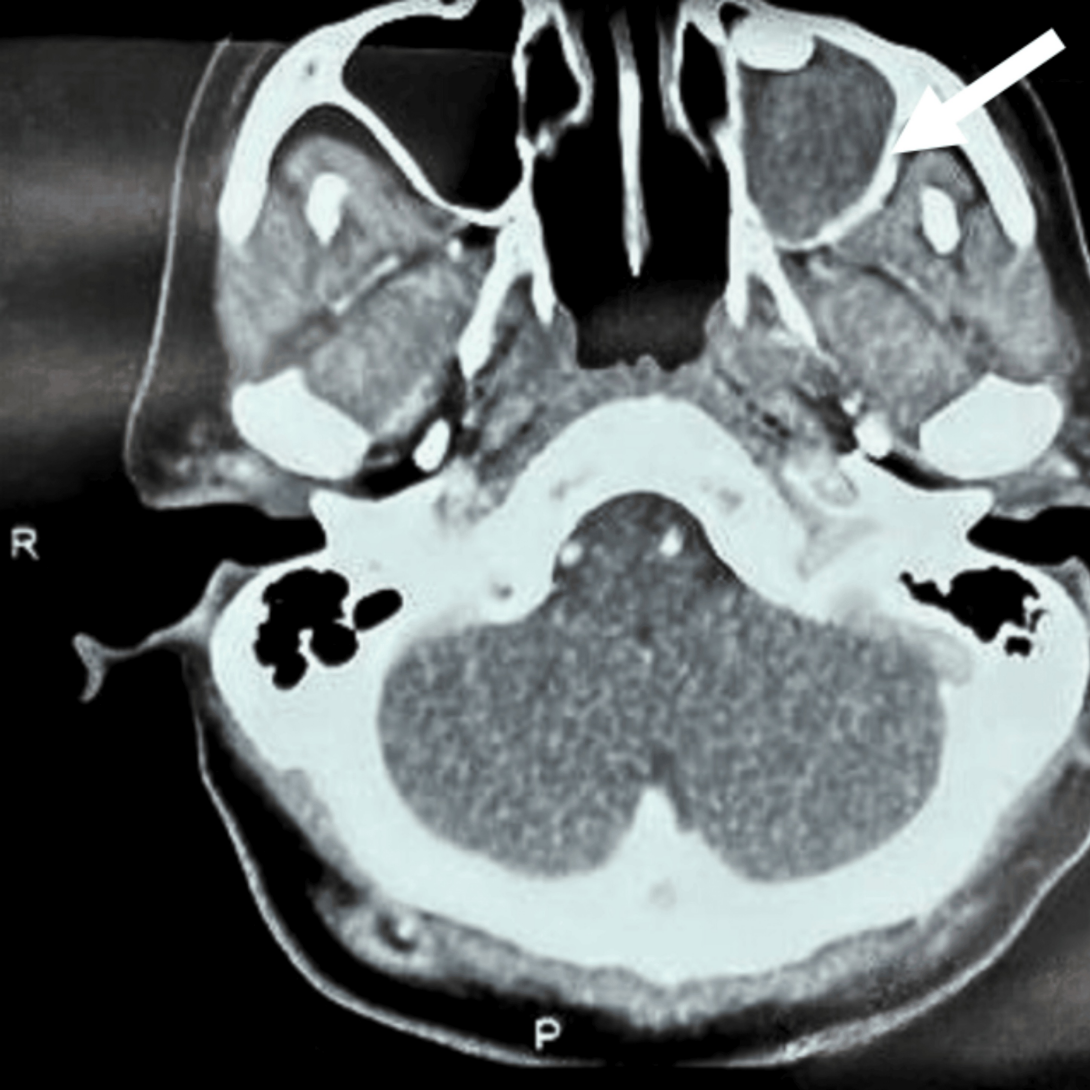
The CT scan (axial view) shows cortical defects over the lateral (white arrow) and inferior aspects of the left maxillary sinus.

A contrasted computed tomography (CT) scan of the face and neck was done (Figures 3-4), which revealed a left maxillary sinus complex cystic lesion occupying the whole left maxillary sinus, causing expansion of the sinus cavity measuring 3.6 x 3.9 x 5.4 cm (width x anteroposterior x height). The mass extended to the left orbital floor superiorly and to the left hard palate inferiorly. Medially, the mass extended to the medial maxillary sinus wall, and laterally, the extension could be seen until the left lateral wall of the maxillary sinus. There was a deformed tooth-like structure at the superior margin of the lesion, with the presence of cortical defects over the lateral and inferior aspects of the left maxillary sinus. However, the left orbital floor appeared intact, sparing the intraorbital extension. A biopsy of the palatal mass performed at a different hospital revealed findings consistent with multicystic ameloblastoma, in keeping with the findings from the CT images.

**Figure 5:**
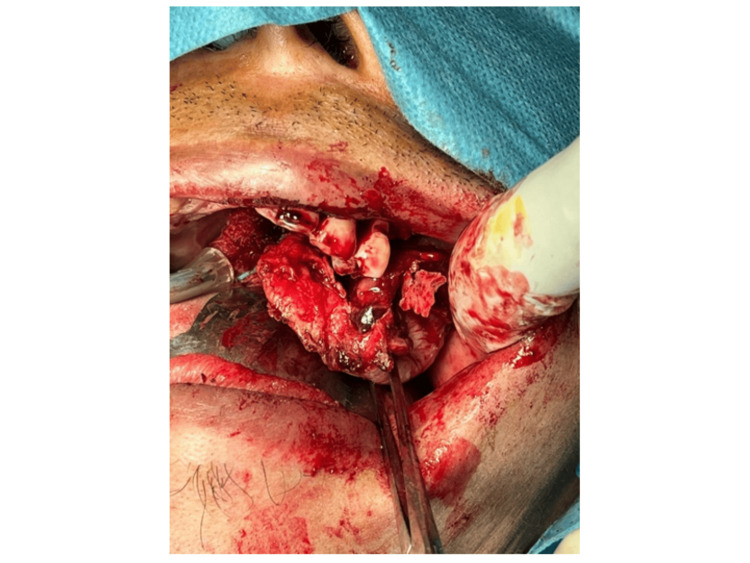
Left maxillary sinus exploration and left partial maxillectomy

**Figure 6:**
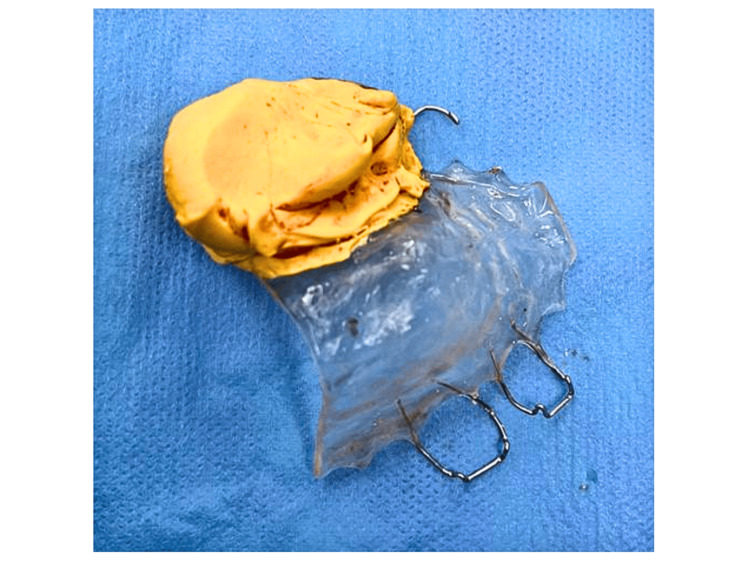
The dressing plate preoperatively prepared with intraoperative adjustment

**Figure 7:**
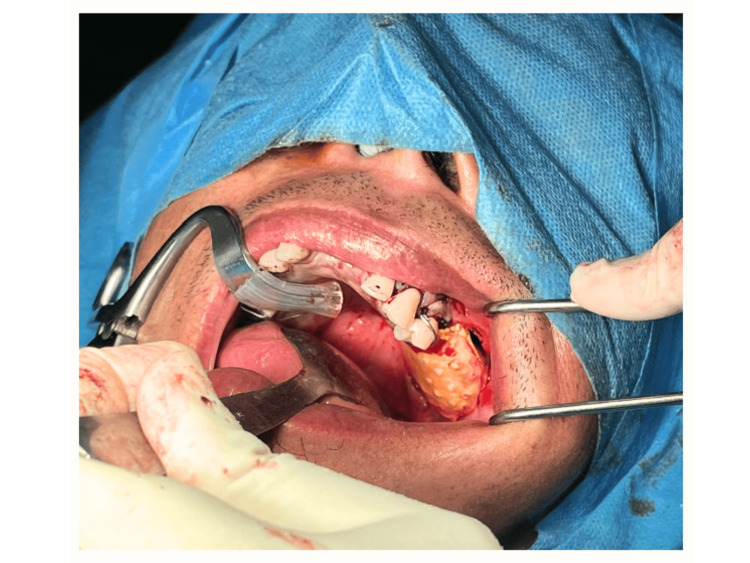
The dressing plate used to cover the defect temporarily

The patient was subsequently referred to our centre for further surgical management of the malignancy, for which a left maxillary sinus exploration and left partial maxillectomy via perioral incision were performed on the patient (Figure 5). The endoscopic approach was not considered as it may not allow en-bloc removal of the tumour. This approach allowed exposure via alveolectomy, palatectomy, and infrastructure maxillectomy. There were no intraoperative complications encountered, and the tumour was completely excised. A dressing plate (Figure 6) was placed in the area of the defect (Figure 7) instead of a flap to allow visualisation of the treated cavity and for surveillance purposes. The patient was started on intravenous (IV) Augmentin (amoxicillin and clavulanate) 1.2 grams three times a day (TDS), IV parecoxib 40 milligrams twice a day (BD), and IV dexamethasone 8 milligrams BD for three doses only.

**Figure 8:**
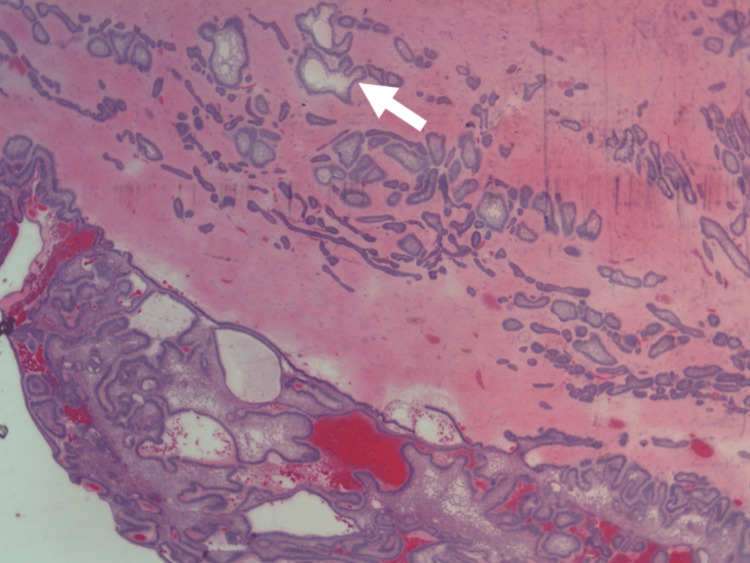
The H&E (20x maginfication) image shows solid tumour stroma containing tumour cells arranged in follicular pattern, cords, and islands. No lymphovascular invasion is identified.

**Figure 9:**
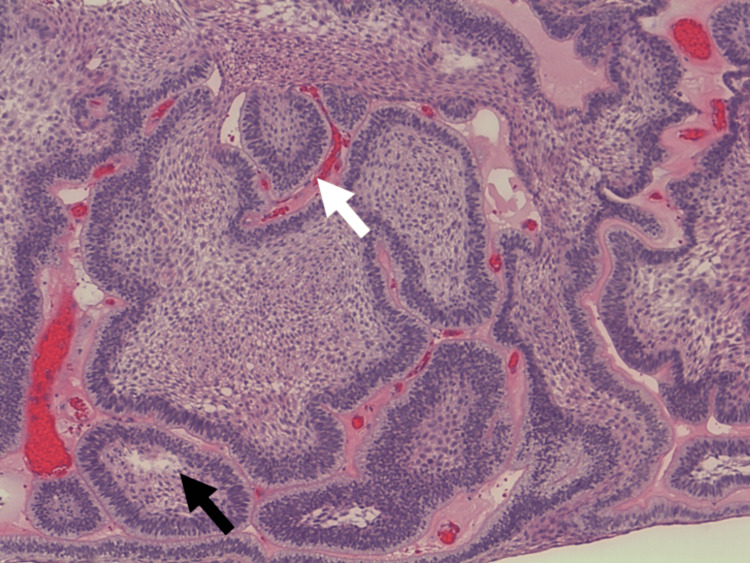
The H&E (100x magnification) image shows ameloblastic epithelium displaying tall columnar cells at the basal layer with hyperchromatic nuclei and peripheral nuclei palisading (white arrow). The suprabasal cells are stellate reticulum-like cells in loose arrangement (black arrow).

**Figure 10:**
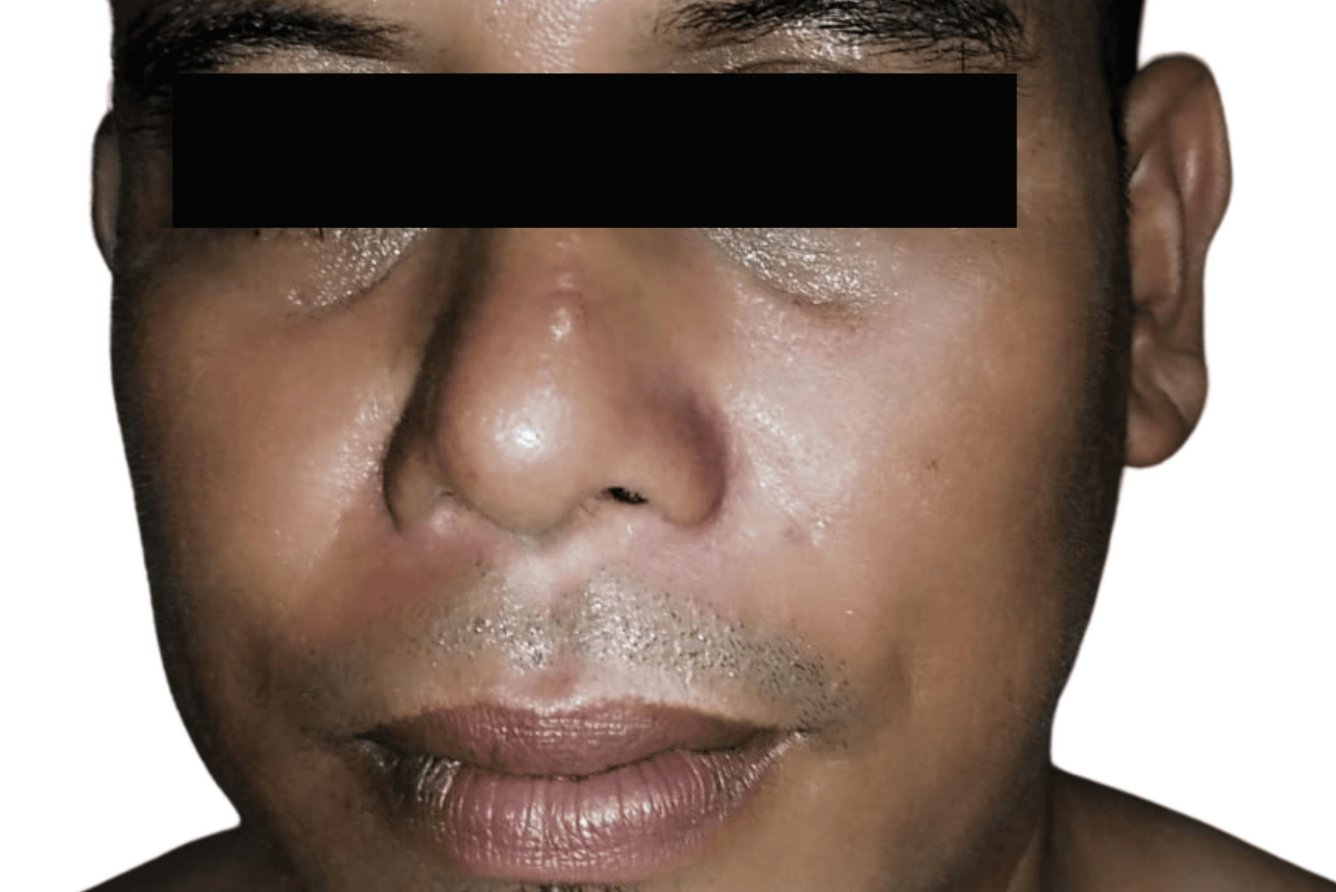
Postoperative progress shows resolved left cheek swelling at one year and six months post surgery.

The haematoxylin and eosin-stained (H&E) sections of the specimen (Figures 8-9) revealed sections of the cystic tumour area lined by ameloblastic epithelium displaying tall columnar cells at the basal layer with hyperchromatic nuclei and peripheral nuclei palisading. The suprabasal cells were stellate reticulum-like cells in loose arrangement with the pericystic solid tumour stroma and showed the presence of tumour cells arranged in follicular patterns, cords, and islands. These tumours also displayed columnar basal cells with peripheral nuclear pallisading and suprabasal stellate reticulum-like cells. There was, however, no lymphovascular invasion identified. The intervening stroma showed fibrohyalinized changes and the presence of mature bone tissue and several lobules of mucus salivary gland acini. The mucosa was covered by stratified squamous epithelium, which shows no dysplasia. This histopathological feature corresponded to ameloblastoma with basal cell features (AM-BC). The tumour cells were reported to be located 2 mm from the anterior, medial, and inferior margins, 3 mm from the posterior and superior margins, and 6 mm from the lateral margin. The patient’s dressing plate was relined with a cold cure on postoperative day four and discharged home. Upon his clinic appointment one month later, he complained of the ill-fitting plate, and necessary adjustments were made as an outpatient. He received his obturator four months after his surgery and is now under yearly surveillance by his referring surgeon. The patient reported having resolved his symptoms with no evidence of recurrence at one year and six months postoperatively (Figure 10).

## Discussion

**Figure 11:**
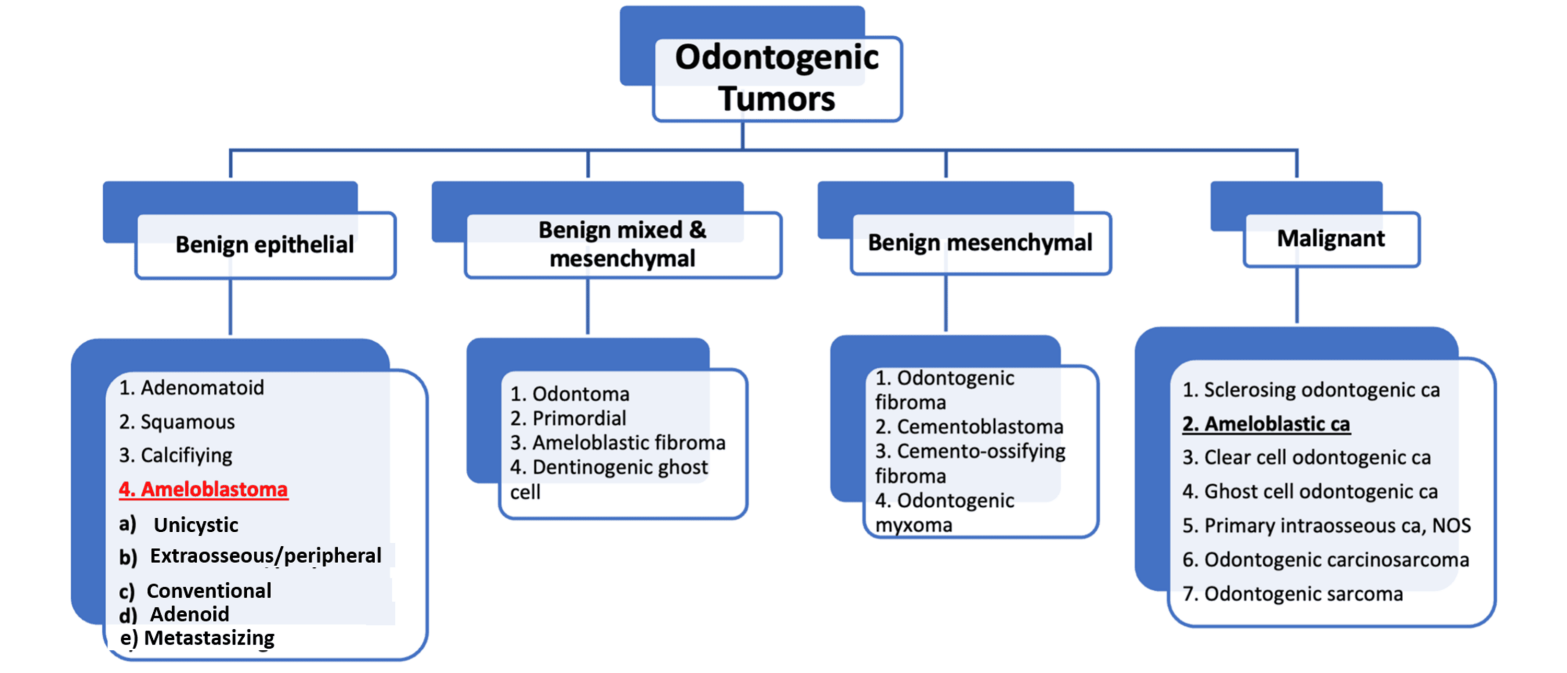
Types of odontogenic tumours Source: [5] ca: carcinoma; NOS: not otherwise specified

Ameloblastoma is the most common odontogenic tumour type, with an incidence of between 35% and 63.1% in multiple studies [8-10] out of the 1.2% diagnosed oral lesions [9]. The most recent WHO Classification of Odontogenic Lesions 2022 (5^th^ Edition) changed the previous classification of metastasising ameloblastoma from malignant to a benign subtype (Figure 11). In view of the primary and metastatic ameloblastomas being similar histopathologically to benign ameloblastomas [5].

A local study of 340 cases of ameloblastoma reported a mean onset age of 30.3 ± 16.3 years [11], which correlates with other studies stating the second and fifth decade, with a majority in their third to fourth decade of life in Asian demographics [8, 9, 12, 13]. Siar et al. [11] also found that the male-to-female ratio is 1.4:1, which shows a slight male predominance compared to another study in the same region, which reported a 1:1.06 male-to-female ratio [14]. This case is one of the 20-36% ameloblastomas of maxillary origin [8, 9], and referring to Figure 2, it can be observed to arise from the posterior maxilla, a more common origin compared to the anterior maxilla, of which only 2%-17% of cases have been reported up to the year 2024 [13, 15]. Maxillary ameloblastoma has also been known to present up to 12 years later than those with mandibular ameloblastoma [13, 16]. This can be due to the patients being asymptomatic [16], and it is challenging to view the lesion clinically and also on plain radiographs [17]. Maxillary ameloblastoma is the more aggressive type compared to mandibular ameloblastoma, primarily due to the invasive nature attributed to the maxilla being a cancellous bone. This, unfortunately, provides more opportunity for the tumour to invade its surrounding structures, namely the nasal cavity medially, maxillary sinus wall laterally, the orbit and skull base superiorly, and the oral cavity inferiorly [18]. Common clinical signs reported by Smit et. al. include swelling (97.1%), pain (27.9%), slow-growing mass (27.2%), mobile teeth (11%), ulcers (6.6%), pus (3.7%), and paraesthesia (2.9%), which this patient exhibited all except mobile teeth, ulcers, and pus [19, 20]. Diagnostic investigations for ameloblastoma include radiological methods such as x-rays, which may show a “soap bubble” appearance; computed tomography scans, which are most commonly done; and magnetic resonance imaging, especially important in cases of maxillary tumours, to assess extension to paranasal sinuses, orbit, or skull base [21]. Trucut biopsy or incisional biopsy is still the gold standard for the diagnosis of ameloblastoma, with a reported accuracy of 88.9% in the oral biopsy result [22].

Genetic testing for the BRAF V600E mutation may also be done for the specimen, as it is found in 70.49% of ameloblastoma cases with a significant association for patients aged less than 54 years old and in cases with mandibular origin [23], although it, unfortunately, won’t be able to predict the patient's prognosis [24]. This will assist patients, especially in the subtype metastatic ameloblastoma, as BRAF inhibitors have been proven to provide good outcomes [25]. Another genetic test that can be conducted is for the smoothened (SMO) gene, which may be present in 14% to 39% of ameloblastoma patients [26, 27]. The SMO gene mutations predominantly occur in the maxilla (57%), whereas BRAF mutations are primarily found in the mandible (75%) [27]. Molecular treatment utilising SMO inhibitors has proven to be less effective due to resistance mechanisms that obstruct the binding of SMO-targeted medicines [26].

Unfortunately, genetic testing is highly costly in local settings, rendering it to be limited only to certain populations with better financial support. Furthermore, if the test will not alter the course of management for the patient, it is usually not performed in cases with a definitive diagnosis.

The primary treatment for this tumour is surgical resection with a negative margin of up to 1 centimetre, and simple curettage must be avoided as it carries a 90%-100% risk of possible recurrence [28, 29]. Tumour surveillance for patients with no presenting symptoms is recommended to be done every six months for the first year and then annually for the next 10 years in the form of orthopantomograms, although CT scans for maxillary ameloblastoma as in this patient are recommended in view of overlapping structures in the area [20]. Since this patient is using a dental obturator, a clinical examination of the previous tumour site must also be done during each follow-up appointment.

## Conclusions

Ameloblastoma, while benign, demands immediate surgical intervention because of its aggressive nature and potential for local invasion. This case emphasises the necessity of a complete clinical and radiographic evaluation in the identification and treatment of maxillary ameloblastoma, especially given its asymptomatic nature and delayed presentation. Regular follow-up and surveillance are critical for detecting recurrence, particularly in patients with maxillary involvement, where the risk of recurrence and sequelae is higher due to the anatomical complexity of the area. The use of dental obturators post surgery helps manage the defect left after the tumour excision and eases direct visualisation for surveillance purposes.
